# BRE plays an essential role in preventing replicative and DNA damage-induced premature senescence

**DOI:** 10.1038/srep23506

**Published:** 2016-03-22

**Authors:** Wenting Shi, Mei Kuen Tang, Yao Yao, Chengcheng Tang, Yiu Loon Chui, Kenneth Ka Ho Lee

**Affiliations:** 1 Stem Cell and Regeneration Thematic Research Programme, School of Biomedical Sciences, Chinese University of Hong Kong, Hong Kong, People’s Republic of China; 2 Department of Chemical Pathology, Chinese University of Hong Kong, Hong Kong, People’s Republic of China

## Abstract

The BRE gene, alias BRCC45, produces a 44 kDa protein that is normally distributed in both cytoplasm and nucleus. In this study, we used adult fibroblasts isolated from wild-type (WT) and BRE knockout (BRE^−/−^) mice to investigate the functional role of BRE in DNA repair and cellular senescence. We compared WT with BRE^−/−^ fibroblasts at different cell passages and observed that the mutant fibroblasts entered replicative senescence earlier than the WT fibroblasts. With the use of gamma irradiation to induce DNA damage in fibroblasts, the percentage of SA-β-Gal^+^ cells was significantly higher in BRE^−/−^ fibroblasts compared with WT cells, suggesting that BRE is also associated with DNA damage-induced premature senescence. We also demonstrated that the gamma irradiation induced γ-H2AX foci, a DNA damage marker, persisted significantly longer in BRE^−/−^ fibroblasts than in WT fibroblasts, confirming that the DNA repair process is impaired in the absence of BRE. In addition, the BRCA1-A complex recruitment and homologous recombination (HR)-dependent DNA repair process upon DNA damage were impaired in BRE^−/−^ fibroblasts. Taken together, our results demonstrate a role for BRE in both replicative senescence and DNA damage-induced premature senescence. This can be attributed to BRE being required for BRCA1-A complex-driven HR DNA repair.

Cellular senescence is an irreversible physiological process that accompanies abnormal and reduced metabolic activities. It is characterized by the loss of proliferation ability of somatic cells such as fibroblasts with some ageing-associated phenotypes^1^. Studies have shown that cellular senescence is closely related to mammalian ageing^2^ and tumor suppression^3^. Clearance of senescent cells can delay or prevent tissue dysfunction and extend lifespan^4^.

Senescence process is complex and derived from different mechanisms. Telomere shortening is a major intrinsic reason. When the telomeres reach a limit called “Hayflick limit”, the cell can no longer divide and becomes senescent or it dies^5^. Cellular senescence can also be induced prior to the stage of telomere depletion by various external conditions. This type of senescence is called “premature cellular senescence”. It is known that persistence of unrepaired double-strand DNA breaks (DSBs) despite activation of DNA damage response (DDR) are the common feature of cellular senescence^6^,^7^. DNA damage commonly occurs during normal metabolic activities^1^, or after exposure to environmental factors of genotoxic potential such as UV light and ionizing irradiation (IR)^8^. A proper response to DNA damage is critical in maintaining genome stability and preventing accumulation and transmission of damaged DNA during cell division. DSBs is a major type of DNA damage lesion and also a powerful activator of DDR. DDR activates cell cycle checkpoints, activates DNA repair pathways, and even initiates apoptosis when the damage level is severe enough. When DDR is initiated, two protein kinases ATR and ATM will be activated to cause the phosphorylation of histone H2AX, which is a key event in DDR. The γ-H2AX (the phosphorylated form of H2AX) is localized around the DSBs, where it is K63-polyubiquitinated by RNF8 and Ubc13. This event leads to the recruitment of a DNA-repair complex, BRCA1-A, to these sites through the interaction between the K63 polyubiquitin chains and RAP80, which is the ubiquitin-binding subunit of the complex^9^,^10^. Defects in DNA repair network cause accumulation of DNA damage and are associated with senescence or carcinogenesis^11^,^12^.

*BRE*, also known as *BRCC45*, was firstly identified as a responsive gene to DNA damage and retinoic acid^13^. It encodes a 383-amino-acid protein which has no identifiable functional domain, with a molecular size of 44 kDa. BRE is located in both cytoplasm and nucleus, with different functions. In the nucleus, BRE has been identified as a member of the BRCA1-A complex, which contains BRCA1-BARD1 heterodimer, RAP80, BRCC36, BRE, ABRA1 and MERIT40. BRE interacts with MERIT40 and is responsible for maintaining the integrity of BRCA1-A complex^14^. It is also required for the K63 deubiquitinase activity of BRCC36 in the complex^15^. The interaction between BRE and BRCC36 has been reported to enhance the E3 ligase activity of BRCA1-BARD1 in a BRCC complex^16^. Knockdown of BRE by siRNA has been shown to increase the sensitivity of cells to IR^14^,^16^. BRE is also a component of multiprotein BRISC (BRCC36 isopeptidase complex) in the cytoplasm. BRISC, comprising BRE, MERIT40, BRCC36 and ABRO1, specifically cleaves K63-specific polyubiquitin chains^17^. Besides, BRE also acts as a death receptor-associated anti-apoptotic protein. It can bind to the cytoplasmic region of Fas^18^, TNF-R1^19^, and death-inducing signaling complex (DISC)^20^ to protect cells from apoptosis.

Since DDR signaling plays important roles in cellular senescence and BRE has been identified as a component of BRCA1-A complex, we set out to investigate the role of BRE in DDR and cellular senescence. In this study, we opted to use normal fibroblasts derived from BRE-knockout mice, instead of cell lines and RNAi-mediated knockdown as reported in previous studies, for our *in vitro* model to demonstrate a role of BRE in BRCA1-driven homologous recombination (HR) DNA repair, replicative senescence and DNA damage-induced premature senescence. To our knowledge, this is the first time that the roles of BRE have been investigated in normal cells with complete depletion of BRE by gene knockout.

## Results

### Generation and characterization of BRE knockout mice

**Figure 1:**
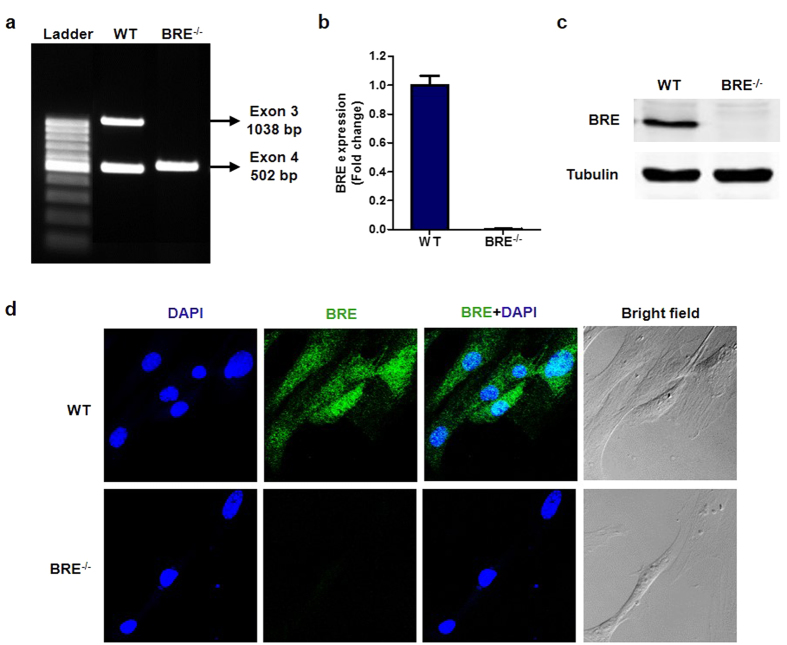
Validation of BRE null mutation in fibroblasts isolated from BRE knockout mice. (**a**) PCR-based genotyping of WT and BRE^−/−^ fibroblasts showing homozygous deletion of *BRE* exon 3 specifically in the latter. (**b**) BRE mRNA expression in WT and BRE^−/−^ fibroblasts as determined by RT-qPCR. House-keeping gene *GAPDH* was used for normalization. (**c**) BRE protein expression in WT and BRE^−/−^ fibroblasts by Western blotting with α-tubulin as protein loading control. (**d**) Immunofluorescence staining of WT and BRE^−/−^ fibroblasts using anti-BRE antibody. Nuclei were counterstained with DAPI. BRE was expressed in both the cytoplasmic and nuclear compartments of WT fibroblasts, but none in the BRE^−/−^ fibroblasts.

To gain insights into some of the functions of BRE in as close to the physiological setting as possible, we chose fibroblasts from adult mouse tail tip instead of long-term laboratory cell lines as our experimental model. We derived the fibroblasts from WT and BRE-knockout mice, which were generated by crossing female BRE^fx/fx^ mice with male TNAP^Cre/+^ transgenic mice as previously described^21^. The BRE^−/−^ mice were normal in appearance; both the male and female mice were fertile. The fibroblasts of the BRE^−/−^ mice showed deletion of the floxed coding exon 3 of BRE in genomic PCR (Fig. 1a). As shown in the figure, the knockout allele yielded no amplified band. The result of real-time (RT) qPCR, which amplified a transcript region spanning the boundary of exons 7 and 8, confirmed that productive *BRE* transcript expression was undetectable in BRE^−/−^ fibroblasts (Fig. 1b). Furthermore, Western blot and immunofluorescence staining verified that the BRE protein was not expressed in these BRE^−/−^ fibroblasts (Fig. 1c,d).

### Effect of BRE deficiency on cell proliferation and cell cycle

**Figure 2:**
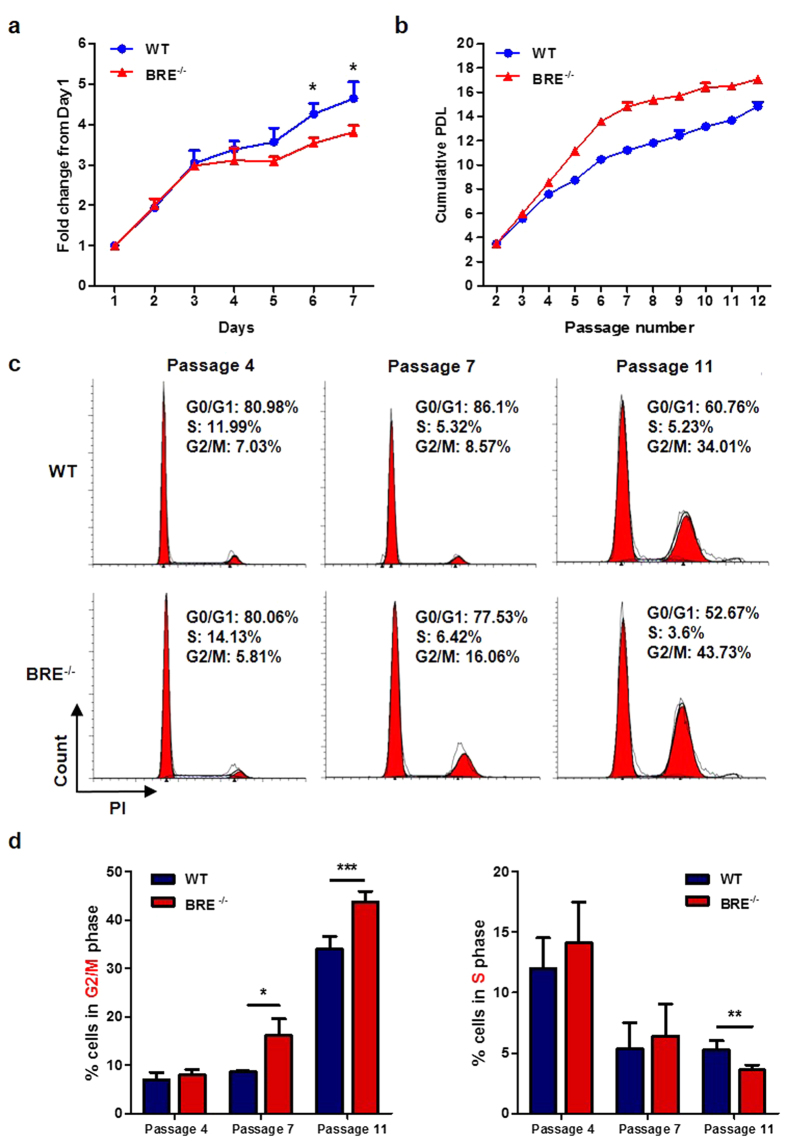
Absence of BRE causes impaired cell proliferation and cell cycle. (**a**) Proliferation curves of WT and BRE^−/−^ fibroblasts expressed in fold change from day 1 when the cultures were initiated. Cell numbers were determined by proliferation assay at the indicated days. Asterisk denotes statistical significance. (**b**) Cumulative population doubling levels (PDL) of WT and BRE^−/−^ fibroblasts passaged every 3 days. (**c**) Cell cycle profiles of WT and BRE^−/−^ fibroblasts at indicated passage numbers. Cells were stained with PI and analyzed by flow cytometry. (**d**) The distribution of cells in G2/M and S phase shown in c is quantified as percentage values. Data shown represent the mean ± SD of three independent experiments. *denotes *P-value* < 0.05, **P-value* *< 0.01 and ***P-value* *< 0.001 for the difference between the WT and BRE^−/−^ fibroblasts.

WT and BRE^−/−^ fibroblasts in culture condition were counted for 7 consecutive days. Compared with WT fibroblasts, the BRE^−/−^ fibroblasts showed reduced proliferation after day 3 (Fig. 2a). Fibroblasts were also subjected to 3T3 protocol for calculation of the population doubling level (PDL). As shown in Fig. 2b, the proliferation rates of WT and BRE^−/−^ fibroblasts were similar in young passages (before passage 4), but in later passages BRE^−/−^ fibroblasts began senescing as indicated by the flattening out of the growth curve starting from passage 7. To determine whether BRE loss could have any effect on cell cycle, we examined the cell cycle profile of WT and BRE^−/−^ fibroblasts at passage 4,7,11 by flow cytometry of propidium iodide (PI) staining (Fig. 2c). Compared with WT fibroblasts, a larger proportion of BRE^−/−^ fibroblasts accumulated 4C DNA content: 8.57% in WT vs. 16.06% in BRE^−/−^ fibroblasts at passage 7, and 34.01% in WT vs. 43.73% in BRE^−/−^ fibroblasts at passage 11. This finding indicates that more fibroblasts remained at the G2/M phase due to slower cell proliferation in the absence of BRE (Fig. 2d). These results provide evidence that BRE effects proliferation and cell cycling.

### Loss of BRE causes DNA damage and accelerated cellular senescence

**Figure 3:**
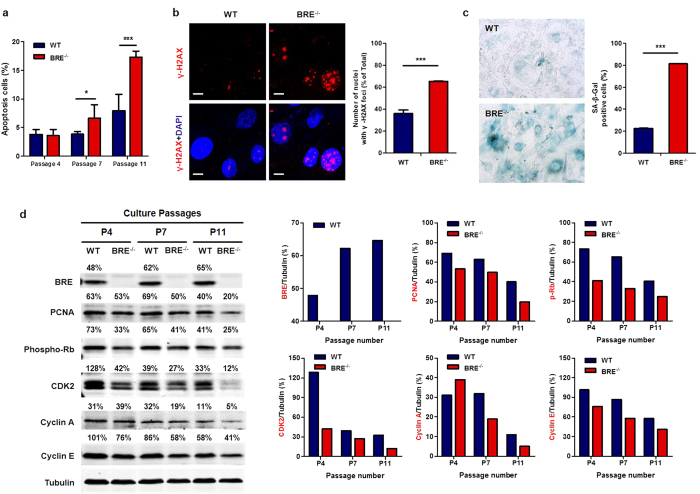
Absence of BRE promotes DNA damage and cellular senescence. (**a**) Percentage of apoptotic cells in WT and BRE^−/−^ fibroblast cultures at indicated passage numbers. Apoptosis was determined by flow cytometry using Annexin V-FITC and PI. Data shown represent the mean ± SD of three independent experiments. *denotes *P-value *< 0.05, and ***P-value < 0.001 for the difference between the WT and BRE^−/−^ fibroblasts. (**b**) Immunofluorescence staining for γ-H2AX foci of WT and BRE^−/−^ fibroblasts at passage 11 (left panel). Scale bars = 10 μm. Percentage of nuclei with more than five γ-H2AX (right panel). At least 150 cells were counted in no fewer than five fields of duplicate plates. Data shown represent the mean ± SD. ***denotes *P-value *< 0.001 versus WT. (**c**) Passage 11 WT and BRE^−/−^ fibroblasts were stained for senescence-associated β-Gal (SA-β-Gal). At least 500 cells were counted in no fewer than five fields of duplicate plates. Data shown represent the mean ± SD. ***denotes *P-value *< 0.001 versus WT. (**d**) Western blot analysis of the expression of BRE and cell cycle regulators PCNA, phosphorylated-Rb (Ser807/811), CDK2, Cyclin A and Cyclin E in WT and BRE^−/−^ fibroblasts at passage number 4, 7 and 11 with α-Tubulin as protein loading control. The expression level of each protein relative to that of α-tubulin is shown as a percentage value on top of its respective image. The bar charts showing the relative expression levels were indicated in the right panel.

Annexin V-FITC and PI apoptosis assay was performed to determine the presence of apoptotic cells in WT and BRE^−/−^ fibroblasts at passage 4, 7 and 11 by flow cytometry. Significantly more spontaneous apoptosis was found among the BRE^−/−^ than WT fibroblasts starting from passage 7: 3.83% in WT vs. 6.62% in BRE^−/−^ fibroblasts at passage 7, and 7.9% in WT vs. 17.33% in BRE^−/−^ fibroblasts at passage 11 (Fig. 3a). The presence of γ-H2AX foci, which is a marker of DNA damage, was also examined by immunofluorescence staining. Spontaneous γ-H2AX foci were found to have increased significantly in BRE^−/−^ fibroblasts compared with WT fibroblasts at passage 11 (36.2% in WT vs. 65.3% in BRE^−/−^ fibroblasts) (Fig. 3b). As induction of DDR signaling is associated with cellular senescence, senescence-associated β-galactosidase (SA-β-Gal) staining was also performed to these fibroblasts. Indeed, the BRE^−/−^ fibroblasts contained significantly more SA-β-Gal positive senescent cells than did the WT fibroblasts: 22.4% in WT vs. 81.5% in BRE^−/−^ fibroblasts, suggesting that the BRE^−/−^ fibroblasts were more prone to senescence (Fig. 3c). Next, the expression of several cell cycle-related proteins at different passage numbers was examined by Western blotting. Expression of BRE was found to increase slightly with the number of passages in the WT fibroblasts, suggesting that BRE may be involved in senescence process. Expression of proliferation markers, PCNA and pRb (phospho-Rb), as well as cell cycle proteins, CDK2, Cyclin A, Cyclin E, which control G1/S and G2/M checkpoint transitions, decreased in both WT and BRE^−/−^ fibroblasts during their passages. However, the decrease was greater in BRE^−/−^ fibroblasts than in WT cells, especially by passage 11 and more markedly so for Cyclin A (Fig. 3d). This result is consistent with the observations above that absence of BRE reduced cell proliferation and increased G2/M cells during passages. Thus, loss of BRE is associated with increased DNA damage and DDR, leading to accelerated cellular senescence.

### Role of BRE in DNA damage response

**Figure 4:**
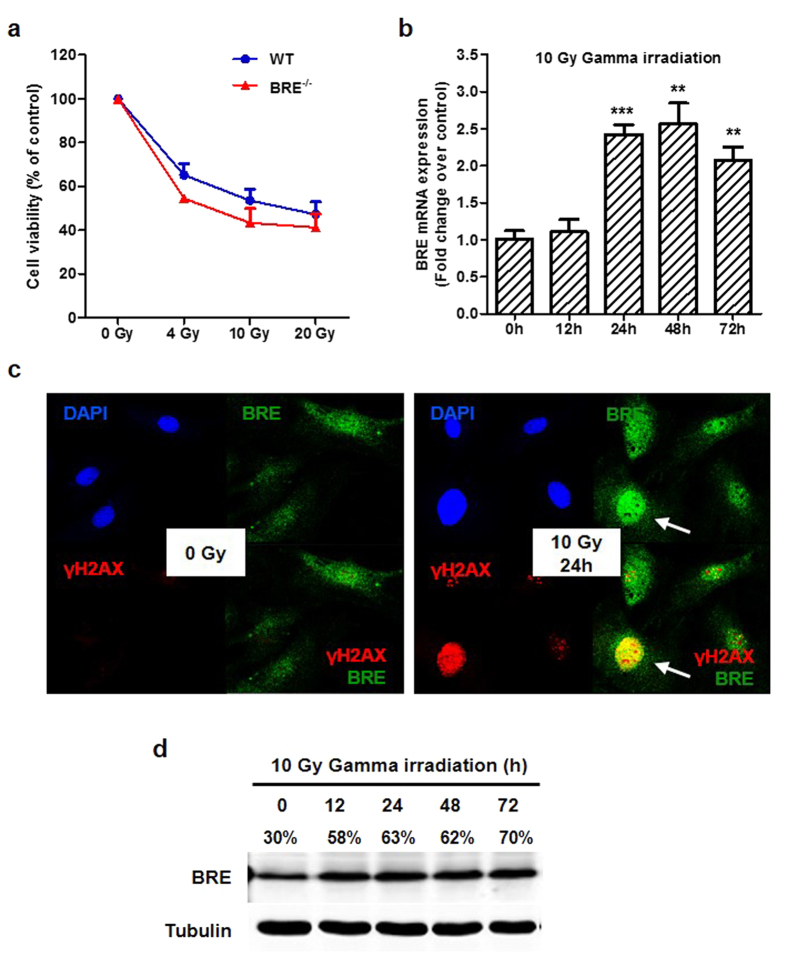
BRE is responsive to DNA damage. (**a**) Viability of early passage WT and BRE^−/−^ fibroblasts in response to a dose range of gamma irradiation. Percentage cell viability was determined using CCK-8 assay on day 7 after each indicated irradiation dose. Shown here is the representative result of fibroblasts at passage number 4. Data represent the mean ± SD of thee independent experiments. (**b**) BRE mRNA levels at indicated time points after 10 Gy of gamma irradiation as determined by RT-qPCR. Data represent the mean ± SD of triplicate wells. **denotes *P-value *< 0.01, and ***denotes *P-value *< 0.001 for the difference compared with 0 h. (**c**) Immunofluorescence staining with anti-BRE antibody (green) and anti-γ-H2AX antibody (red), together with DAPI nuclear staining (blue), of WT fibroblasts at 24 h after 10 Gy of gamma irradiation compared with the control without irradiation. Each panel comprises 4 images of the same field of cells showing the staining by DAPI (top left), γ-H2AX (bottom left), BRE (top right) and the composite of γ-H2AX and BRE (bottom right). Arrowheads indicate examples of increased nuclear BRE expression in association with DNA damage. (**d**) Western blot analysis for BRE expression of WT fibroblast whole cell lysates at indicated time points after 10 Gy of gamma irradiation. Relative expression levels of BRE versus α-tubulin are shown as percentage values.

On the basis of the above data, we postulated that the accelerated cellular senescence observed in BRE^−/−^ fibroblasts is attributed to the accumulation of DNA damage which cannot be effectively repaired as the cells are proliferating. To verify the hypothesis, we induced DNA damage in WT and BRE^−/−^ fibroblasts between passages 3 through 5 (early passages) by exposing these cells to 4, 10 and 20 Gy of gamma irradiation, and compared their radiosensitivity. It was found that the viability of both WT and BRE^−/−^ fibroblasts decreased in a dose-dependent manner, with the latter showing a trend of larger decrease although the differences were not statistically significant (Fig. 4a). We chose 10 Gy of irradiation to study the radioresponsive response of BRE in the WT fibroblasts. RT-qPCR showed that the mRNA expression of BRE was significantly upregulated from 24 h after irradiation, and remained elevated at 48 h (Fig. 4b). Immunofluorescence staining revealed the presence of γ-H2AX foci and marked increase in the expression of BRE in the nuclei at 24 h after irradiation, indicating that BRE was responsive to DNA damage (Fig. 4c). Western blot showed that the protein level of BRE was upregulated from 12 h after irradiation, which was earlier than the increase in mRNA level, perhaps due to stabilization of the protein or enhanced translation (Fig. 4d).

### Loss of BRE enhances DNA damage-induced premature senescence but not affect activation of cell cycle checkpoint

**Figure 5:**
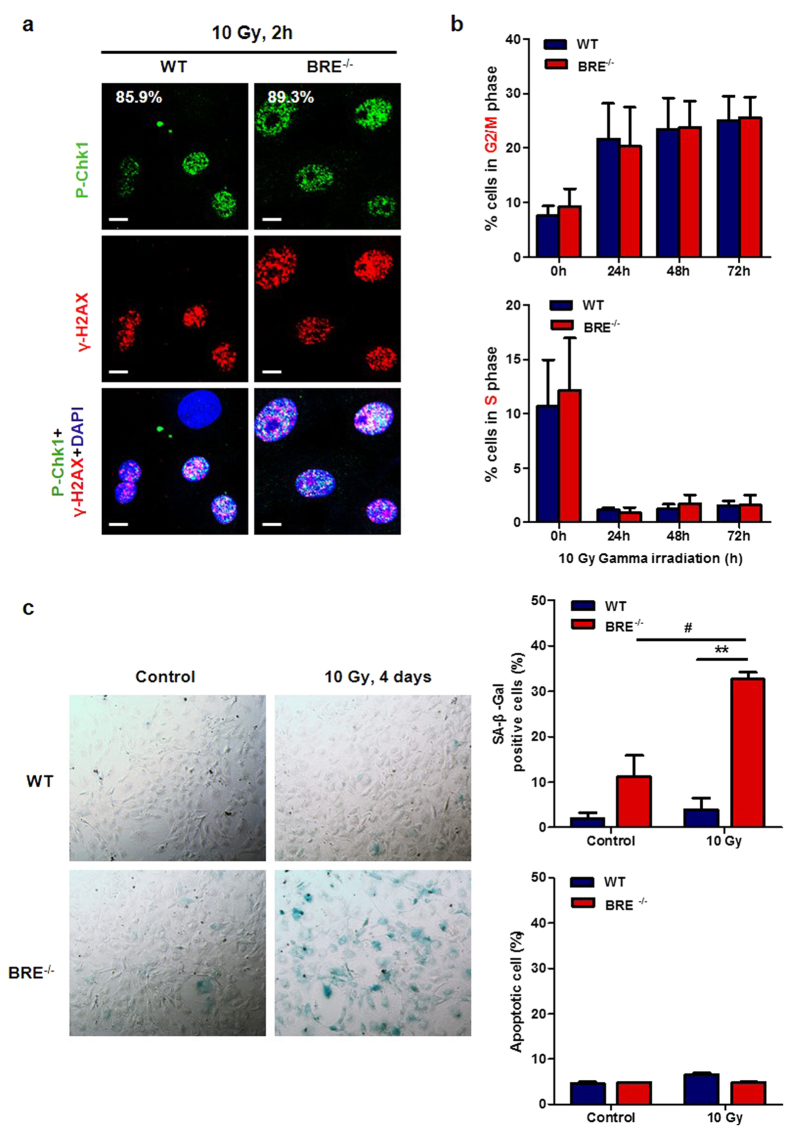
BRE^−/−^ fibroblasts are sensitized to DNA damage-induced premature senescence. (**a**) Immunofluorescence staining of passage 4 WT and BRE^−/−^ fibroblasts at 2 h after 10 Gy of gamma irradiation using anti-phospho-Chk1(P-Chk1) antibody (green) and anti-γ-H2AX antibody (red), together with DAPI nuclear staining (blue). Scale bars = 10 μm. (**b**) Cell cycle distribution of WT and BRE^−/−^ fibroblasts at indicated time points after gamma irradiation. The percentages of cells at G2/M phase (upper panel) and S phase (lower panel) were obtained by PI staining for flow cytometric analysis using ModFit LT software. Data represent the mean ± SD of three independent experiments. No statistical significance between the WT and BRE^−/−^ fibroblasts was found. **(c)** Senescence-associated β-Gal (SA-β-Gal) staining of WT and BRE^−/−^ fibroblasts at 4 days after 10 Gy of irradiation (left panel). The percentage of SA-β-Gal positive cells increased significantly among the irradiated BRE^−/−^ fibroblasts compared with WT fibroblasts (top right panel). At least 500 cells were counted in no fewer than five fields of duplicate plates. Data represent as the mean ± SD. **denotes *P-value *< 0.01 versus WT, ^#^denotes *P-value *< 0.05 versus untreated. The percentages of apoptosis cells in WT and BRE^−/−^ fibroblasts were determined by Annexin V-FITC and PI staining at 24 h after 10 Gy of irradiation (bottom right panel). Data represent the mean ± SD of three independent experiments. No statistical significance was found.

Cell cycle checkpoint regulation is a critical part in DDR, as cell cycle arrest allows time for the damaged DNA to be repaired. DNA damage activates both the G2/M and the G1/S checkpoints. To determine whether loss of BRE would affect checkpoint activation after DNA damage, the phosphorylation level of Chk1 kinase (Ser345), which is an important checkpoint controller downstream of ATM/ATR kinase, was examined by immunofluorescence. Chk1 was found to be similarly phosphorylated and co-localized with γ-H2AX foci in the nucleus of both WT and BRE^−/−^ fibroblasts, suggesting that absence of BRE does not affect cell cycle checkpoint activation (Fig. 5a). This finding was corroborated by measuring the DNA content distribution of WT and BRE^−/−^ fibroblasts in response to irradiation by flow cytometry of PI staining. At 24 h after 10 Gy of irradiation, both WT and BRE^−/−^ fibroblasts exhibited comparable accumulation of cells in G2/M phase and decrease in S phase (Fig. 5b). While Annexin V-FITC and PI staining showed only low levels of apoptosis, which were less than 10%, to both of the WT and BRE^−/−^ fibroblasts following irradiation, a marked elevation of senescence was detected in the BRE^−/−^ fibroblast population. Staining by SA-β-Gal showed that at 4 days after 10 Gy of irradiation, BRE^−/−^ fibroblasts contained dramatically increased number of SA-β-Gal positive senescent cells than irradiated WT fibroblasts: 3.9% in WT vs. 32.6% in BRE^−/−^ fibroblasts. This result clearly indicates that BRE^−/−^ fibroblasts were more sensitized to DNA damage-induced premature senescence (Fig. 5c). The requirement of BRE for preventing DNA damage-induced premature senescence was also observed in BRE siRNA-transfected fibroblasts at 7 days after irradiation: 29.1% in control vs. 46.9% in BRE-silenced fibroblasts (Supplementary Fig. S1). Another DNA-damaging agent H_2_O_2_ was employed to test the role of BRE in DNA damage-induced premature senescence. The results showed that at 7 days after 200 μM of H_2_O_2_ pre-treatment, BRE^−/−^ fibroblasts also contained a substantially increased number of SA-β-Gal positive senescent cells compared with the WT cells (7.4% in WT vs. 35.1% in BRE^−/−^ fibroblasts) (Supplementary Fig. S2).

### Loss of BRE impairs DNA repair process

**Figure 6:**
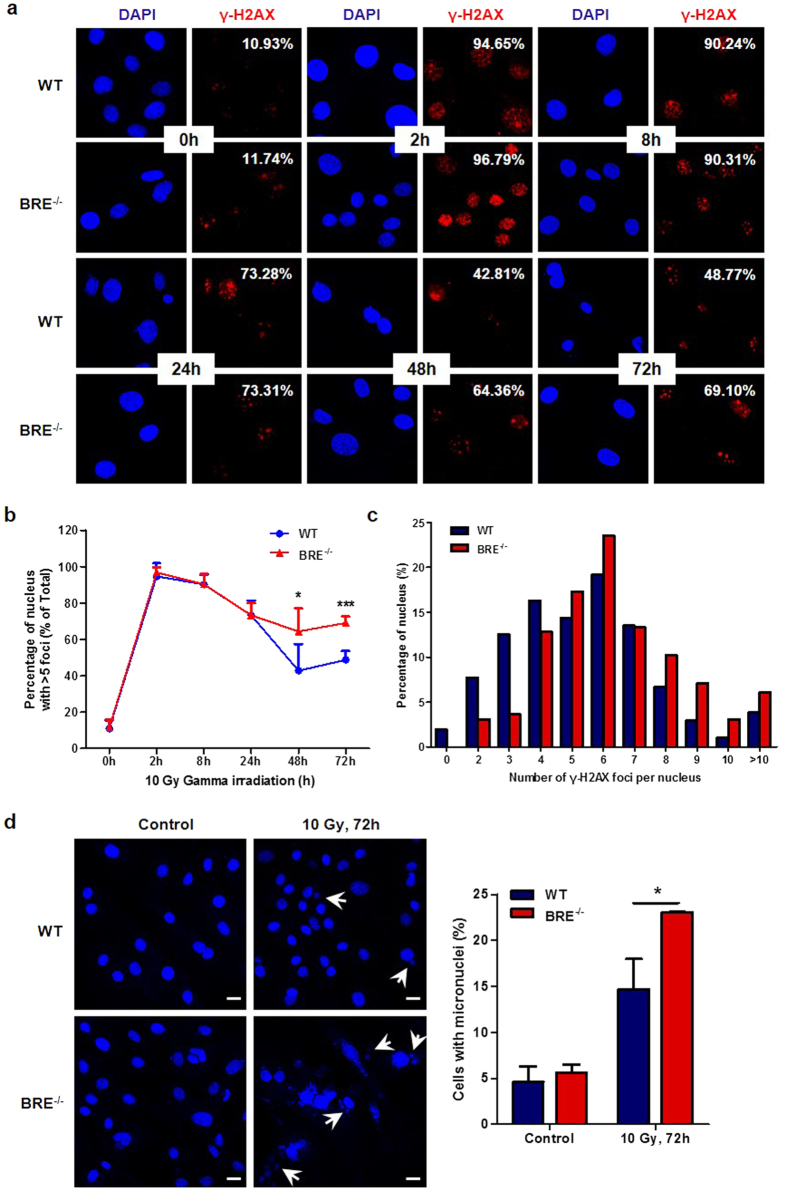
The DNA repair process is impaired in BRE^−/−^ fibroblasts. (**a**) Immunofluorescence staining of passage 4 WT and BRE^−/−^ fibroblasts at indicated time points after 10 Gy of gamma irradiation using anti-γ-H2AX antibody (red), together with DAPI nuclear staining (blue). (**b**) Quantification of nuclei with more than five γ-H2AX foci in percentage at each time point for the WT and BRE^−/−^ fibroblasts above. At least 150 cells were scored for the γ-H2AX foci in no fewer than five fields of duplicate plates. Note the significantly longer persistence of γ-H2AX foci in BRE^−/−^ fibroblasts compared with WT fibroblasts. Data represent the mean ± SD. *denotes *P-value *< 0.05, ***denotes *P-value *< 0.001 versus WT. (**c**) Tabulation of the percentage of nuclei containing indicated numbers of γ-H2AX foci at 72 h after irradiation. (**d**) DAPI nuclear staining showing more cells with fragmented micronuclei among the BRE^−/−^ compared with WT fibroblasts at 72 h after irradiation. Arrowheads indicate examples of micronuclei (left panel). Scale bars = 20 μm. Quantification of cells with micronuclei (right panel). At least 300 were counted in no fewer than five fields of three independent experiments. Data represent as the mean ± SD *denotes *P-value *< 0.05 versus WT.

Our observed association between the absence of BRE and increased baseline as well as DNA damage-induced premature cellular senescence led us to clarify the role of BRE in DNA repair process. WT and BRE^−/−^ fibroblasts were exposed to 10 Gy of gamma irradiation and collected at different time points for γ-H2AX immunofluorescence. At 2 h after irradiation, almost all WT and BRE^−/−^ fibroblasts were strongly positive for γ-H2AX staining, followed by a decline of the percentage of nuclei with more than five γ-H2AX foci in both cell populations. However, the γ-H2AX foci persisted longer in BRE^−/−^ fibroblasts compared with WT fibroblasts, suggesting that the repair of IR-induced DNA damage progresses slower without BRE (Fig. 6a,b). A similar result was obtained using a lower irradiation dose of 4 Gy (Supplementary Fig. S3). The number of γ-H2AX foci per nucleus was also determined at 72 h after 10 Gy of irradiation, BRE^−/−^ fibroblasts contained more γ-H2AX foci than WT fibroblasts (Fig. 6c). In addition, at 72 hours after irradiation, BRE^−/−^ fibroblasts contained increased number of fragmented micronuclei compared with WT fibroblasts (14.7% in WT vs. 23.0% in BRE^−/−^ fibroblasts), suggesting that BRE also associates with genomic instability (Fig. 6d). These data show that the DNA repair is less efficient without the involvement of BRE.

### Loss of BRE impairs BRCA1-A complex recruitment and HR-dependent DNA repair

**Figure 7:**
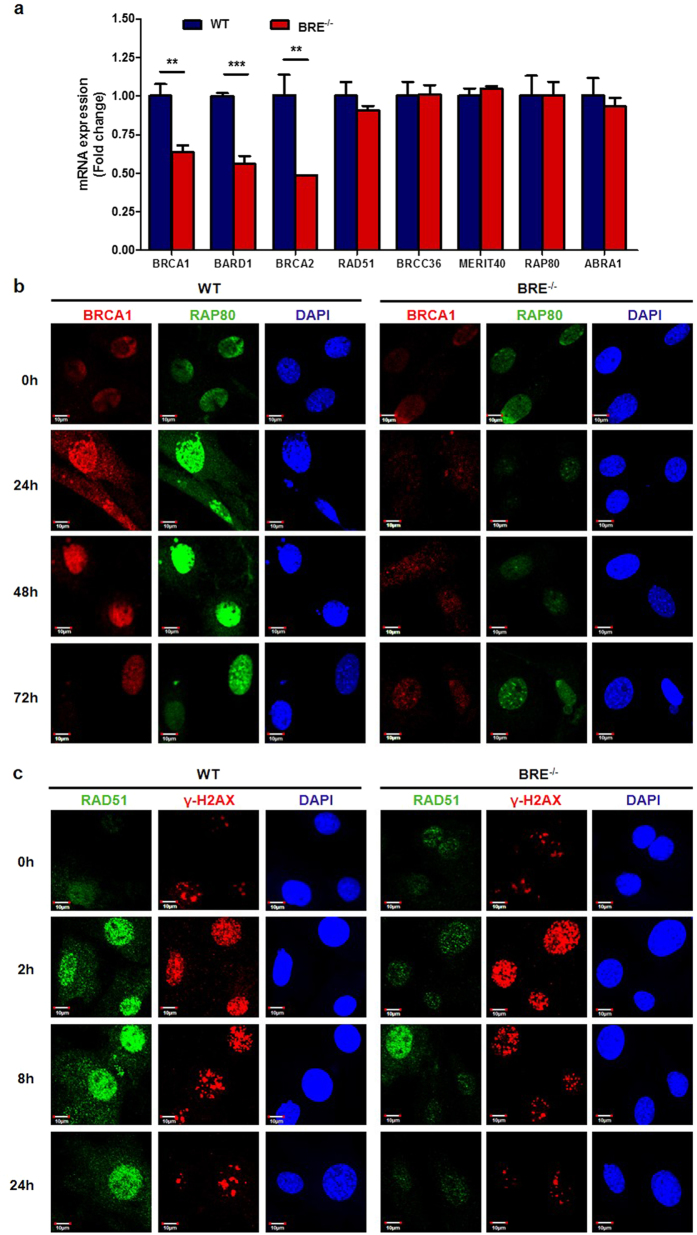
The recruitment of BRCA1-A complex and HR-dependent DNA repair are impaired in BRE^−/−^ fibroblasts. (**a**) mRNA expression of BRCA1-A complex components in WT and BRE^−/−^ fibroblasts as determined by RT-House-keeping gene *GAPDH* was used for normalization. Note the significant downregulation of BRCA1, BRCA2 and BARD1 in BRE^−/−^ fibroblasts. Data represent the mean ± SD of triplicate wells. **denotes *P-value *< 0.01, ***P-value < 0.001 versus WT. (**b**) The foci formation of BRCA1 and RAP80 at 24, 48 and 72 h after 10 Gy of gamma irradiation as examined by immunofluorescence staining, together with DAPI nuclear staining. (**c**) RAD51 and γ-H2AX foci formation by immunofluorescence staining at 2, 8 and 24 h after 10 Gy of gamma irradiation. Scale bars = 10 μm. Note the weaker BRCA1, RAP80 and RAD51 foci staining of BRE^−/−^ fibroblasts compared with the WT.

To investigate how the loss of BRE leads to impairment of IR-induced DNA damage, we first checked the baseline mRNA expression levels of BRCA1-A complex subunits, which include BRCA1, BRCA2, BARD1, RAD51, BRCC36, MERIT40, RAP80 and ABRA1. RT-qPCR revealed that the expression of BRCA1, BRCA2 and BARD1 but not the rest was significantly decreased in BRE^−/−^ fibroblasts compared with WT fibroblasts (Fig. 7a). Immunofluorescence staining showed that in response to 10 Gy of irradiation not only the dynamic foci formation of BRCA1 was dramatically reduced, the recruitment of RAP80 (Fig. 7b) and the DNA recombination protein RAD51 (Fig. 7c), whose transcript levels were not reduced, was also virtually abolished in BRE^−/−^ fibroblasts. Thus, BRE is required for the recruitment of RAP80 to the IR-induced DNA damaged sites. With impaired RAP80 recruitment and reduced BRCA1 expression in the absence of BRE, foci formation by BRCA1 and RAD51 is also diminished.

## Discussion

The mRNA and protein of BRE are ubiquitously expressed in most tissues and cell types. It has been reported that BRE is required for the integrity of BRCA1-A complex in the nucleus and BRISC (BRCC36 isopeptidase complex) in the cytoplasm^14^,^22^. Besides, it also functions in preventing apoptosis^23^ and maintaining stemness^24^. However, these previous findings were based on experimental designs where BRE was either overexpressed or silenced by RNAi, the latter of which could yield results confounded by the presence of a residual level of the protein. We, therefore, generated a BRE knockout mouse model, in which the exon 3 of the gene crucial to expression was deleted. No visible abnormality was observed during embryonic and adult development of these mice. During the study period of which the oldest mice reached about 18 months old, no significant difference in lifespan and tumor incidence was observed in BRE^−/−^ versus the WT mice. However, we found that the adult fibroblasts derived from BRE^−/−^ mice undergo cellular senescence earlier than the WT fibroblasts. This finding is consistent with previous reports that BRCA1- and RAP80-null MEFs are prone to senescence in association with genomic instability^12^,^25^. Cellular senescence is one kind of DDR manifested as irreversible cell cycle arrest, although the cells remain alive. Senescent cells are known to accumulate irreparable DNA double-strand breaks^6^,^7^. Since DNA damage occurs frequently in cells, efficient DDR and repair are important in maintaining cellular genomic stability. In this study, we found that the cultured BRE^−/−^ fibroblasts contained increased number of spontaneous γ-H2AX foci compared with WT fibroblasts at the same passage numbers, suggesting decreased efficiency in repairing the spontaneous single and double strand breaks in BRE^−/−^ fibroblasts cells. Thus, we have provided evidence that BRE functions in the repair of baseline DNA damage, and this ability is associated with the prevention of premature cellular senescence.

Regarding the role of BRE in forming IR-induced foci for repairing DNA damage, our findings using fibroblasts are similar to those previously reported using immortalized cell lines^14^,^26^. However, we found that in contrast to the RNAi-mediated BRE knock-down immortalized cell lines reported by others, our BRE knock-out fibroblasts were not more sensitive to IR-induced cell death than the WT fibroblasts, and their cell cycle G2/M checkpoint was not disrupted^14^,^16^. Our data instead were more similar to those of the mouse embryonic fibroblasts (MEF) derived from RAP80 knockout mice^25^. Like our BRE^−/−^ fibroblasts, both of the RAP80^−/−^ and WT MEFs upon 4 Gy of IR exposure exhibited a marked increase in G2/M cells, indicating that the G2/M checkpoint remains as much functional in the absence of RAP80 as in BRE knockout. Despite the closer comparison between our experimental system and that of the RAP80^−/−^, where gene knockout approach and normal cells (i.e. fibroblasts) were used, there are still several discrepancies between the results of BRE^−/−^ fibroblasts and the RAP80^−/−^ MEF. First, while our BRE^−/−^ and WT fibroblasts showed comparable accumulation of IR-induced G2/M cells, the RAP80^−/−^ MEFs accumulated significantly more of the G2/M cells than the WT cells. This discrepancy, we surmise, could be due to the use of a lower dose of irradiation (i.e. 4 Gy) in the RAP80^−/−^ study, allowing the WT MEFs to exit the G2/M block faster than the DNA repair-defective RAP80^−/−^ cells. By contrast, our use of 10 Gy of irradiation would likely delay the exit of the WT fibroblasts. Second, the RAP80^−/−^ MEFs were significantly more apoptotic than the WT MEFs in response to IR, whereas no elevation of IR-induced cell death was shown by our BRE^−/−^ fibroblasts compared with the WT cells. We note that in the RAP80^−/−^ study when SV40 T-antigen-immortalized RAP80^−/−^ and WT iMEFs (immortalized MEFs) were irradiated, the difference in apoptosis between the 2 cell populations was greatly reduced, due to a marked decrease in apoptosis of the RAP80^−/−^ iMEFs. This increased resistance to IR-induced cell death of the RAP80^−/−^ iMEFs was shown to be resulted from inactivation of p53 by the SV40 T-antigen. Interestingly, we have previously shown that expression of p53 is regulated by BRE in such a way that its level is down-regulated by siRNA-mediated BRE knockdown and up-regulated by BRE overexpression^27^. Thus, the lack of sensitivity of the BRE^−/−^ fibroblasts to IR-induced apoptosis could be due to downregulation of p53. We are at present investigating this possibility, and our preliminary finding indeed suggests a lack of p53 response to IR in the absence of BRE. It should be noted that, instead of apoptosis, senescence was the major response of the BRE^−/−^ fibroblasts to IR. Thus, BRE may also have a hitherto unknown but important p53-independent role in preventing cellular senescence associating with genome instability. One other previously unreported observation in this present study is the downregulation at mRNA level of BRCA1, BRCA2 and BARD1 in the absence of BRE. Although it could not be ruled out that BRE may simultaneously regulate the expression of these 3 genes at transcriptional or post-transcriptional level (e.g. via microRNAs), a more likely explanation is that this simply reflects the effect of BRE knockout on cell cycling. It is conceivable that it was the slower proliferation of BRE^−/−^ fibroblasts rather the absence of BRE per se which led to the lower mRNA levels of the 3 DDR genes, whose expression has been reported to be cell-cycle dependent^28^,^29^,^30^.

Intriguingly, despite the enhanced baseline and stress-induced cellular senescence, and impaired repair of spontaneous double-stranded DNA damage shown by the BRE^−/−^ fibroblasts, the BRE knockout mice displayed neither discernible shortening of lifespan nor increase in spontaneous tumor incidence. This observation is in marked contrast to the mouse models carrying BRCA1, BRCA2 and RAP80 mutation^12^,^25^,^31^,^32^. It remains to be seen whether BRE knockout would render the mice more susceptible to IR-induced senescence and/or tumor formation.

In summary, a BRE knockout mice model was generated, and the functions of BRE in DNA repair as well as senescence were investigated. Our study indicates that BRE is responsible for HR-dependent DNA DSBs repair through facilitating localization of BRCA1-A complex to DNA damage sites. Deletion of BRE leads to accumulation of unrepaired DNA damage, which in turn sensitizes the cells to stress and promotes them to undergo premature senescence.

## Methods

### Generation of BRE knockout mice

The BRE knockout mouse model used in this study was previously published^21^. Briefly, these mice were generated using the Cre/LoxP recombination system and bred into the C57/BL/6J background. The BRE targeted strain (B6Dnk; B6N-Bre^tm1a (EUCOMM) Wtsi/H^), in which the exon 3 of BRE gene was flanked by two loxP sites, was purchased from European Conditional Mouse Mutagenesis Programme (EUCOMM). TNAP-Cre (TNAP^Cre/+^) mice (129-Alpl^tm1 (cre) Nagy^/J, Stock Number: 008569, Jackson Laboratory), which is a PGCs (primordial germ cells)-Cre transgenic mice, was used to cross with female BRE^fx/fx^ mice to generate BRE^−/−^ mice. All animal experiments were approved by AEEC (Animal Experimentation Ethics Committee) of Chinese University of Hong Kong and Hong Kong Government Department of Health. And animal procedures were carried out in accordance with the approved guidelines.

### Establishment of adult mouse fibroblast cultures

Fibroblasts were isolated from tail clips of 3-month old wide-type (WT) and BRE knockout (BRE^−/−^) mice. Briefly, the tail clip was diced into small pieces and placed into a 1.5 mL microcentrifuge tube, to which 0.5 mL of collagenase was added to the final concentration of 1000 U/mL. After incubation at 37 °C for 30 min, followed by centrifugation at 1000 rpm for 5 min, the pellet was collected and treated with 0.5 mL of 0.05% trypsin at 37 °C for 20 min. After centrifugation as above, the digested pellet was collected, resuspended in DMEM/F12 medium containing 10% FBS and transferred into a 10-cm^2^ culture dish for incubation at 37 °C with 5% CO_2_.

### Cell proliferation/ viability assay

WT and BRE^−/−^ fibroblasts were harvested and seeded into 96-well plates at 1 × 10^4^ cells/well. The cells were either left untreated or treated with 10 Gy of gamma irradiation (Nordin Gammacell 3000 Elan). Ten μL of CCK-8 solution (Dojindo) was added to each well at the day of cell counting for incubation for 2 h at 37 °C. Thereafter, OD_450_ was measured using a microplate reader (BIO-RAD Benchmark Plus). Cell proliferation was calculated as the ratio of OD_450_ of cells at indicated day to OD_450_ of cells at day 1. Cell viability was calculated as OD_450_ of treated cells to OD_450_ of untreated cells.

### Examination of cumulative population doubling level (PDL)

WT and BRE^−/−^ fibroblasts were seeded in 6-well plates at a density of 1 × 10^5^ cells/well. Cultures were split every 3 days and re-plated at the same density (i.e. 1 × 10^5^). Cell counting was performed at each passage. The cumulative population doubling level (PDL) of each passage was calculated by the formula PDL = log (n_f_/n_0_)/ log2 + X, where n_0_ is the initial number of cells at the beginning of the subculture, and n_f_ the final number of cells at the end of that subculture. X is the doubling level of the cells at the initiation of the subculture, i.e. the cumulative PDL of the last passage.

### Cell cycle analysis

WT and BRE^−/−^ fibroblasts were either left untreated or treated with 10 Gy of gamma irradiation. At 24, 48 and 72 h after irradiation, the cells were trypsinized, fixed in 70% ethanol and stored at −20 °C for overnight. After washing with PBS, the cells were stained with PI solution (0.04 mg/mL of PI, 0.25 mg/mL of RNase A, 0.1% Triton X in PBS) for 30 min, and then subjected to flow cytometry analysis (BD LSRFortessa Cell Analyzer). DNA content was determined on counting 10,000 cells. Percentage of cells in each cell cycle phase was analyzed using ModFit LT (Verity Software House) software.

### Apoptosis Assay

The percentage of apoptotic cells was determined by flow cytometry using Annexin V-FITC/ Dead Cell Apoptosis Kit (Invitrogen) according to the manufacturer’s protocol. Briefly, cells were harvested and stained with 5 μL of Annexin V-FITC and 1 μL of 100 μg/mL PI solution in binding buffer. Annexin V-positive/PI-negative, Annexin V-positive/PI-positive and Annexin V-negative/PI-positive cells represent cells in early apoptosis, late apoptosis and necrosis respectively.

### Senescence-associated β-galactosidase (SA-β-Gal) staining

The staining was performed as described by Debacq-Chainiaux *et al.*^33^. Briefly, cells were fixed in PBS containing 2% formaldehyde and 0.2% glutaraldehyde for 5 min. After 3 washes with PBS, SA-β-Gal staining solution with 1 mg/mL of X-Gal was added and incubated at 37 °C for overnight. On the second day, the cells were washed with PBS for photomicrography.

### Immunofluorescence and microscopy

WT and BRE^−/−^ fibroblasts were seeded onto coverslips. Cells with or without treatment with 10 Gy of gamma irradiation were fixed with 4% formaldehyde at indicated time points for 1 h and permeabilized with 0.5% Triton X-100 for 30 min at room temperature. After blocking with PBS containing 5% normal goat serum for 1 h, the cells were then incubated with primary antibodies diluted with the blocking solution for overnight at 4 °C. Thereafter, the cells were washed with PBST (0.05% Tween-20 in PBS), followed by incubation with Alexa Fluor-conjugated secondary antibodies diluted in PBST for 1 h at room temperature, counterstained with DAPI (4 μg/mL), and mounted in 70% glycerol. Images were taken under a laser spot scanning confocal system (Olympus FV1000) with 40× and 100× objective lenses and analyzed using FV10-ASW 3.1 viewer software.

The primary antibodies included rabbit anti-BRE (Cell Signaling Technology, 1:200 dilution), mouse anti- phospho-Histone H2AX (γ-H2AX, Ser139) (Upsate, 1:500), rabbit anti-phospho-Chk1 (Thr345) (Cell Signaling Technology, 1:100), mouse anti-BRCA1 (Thermo Scientific, 1:100), rabbit anti-RAP80 (ProSci Inc, 1:100), rabbit anti-RAD51 (Santa Cruz, 1:100). The secondary antibodies included Alexa Fluor 488 donkey anti-rabbit IgG and Alexa Fluor 647 donkey anti-mouse IgG (Invitrogen, 1:300).

### Western blot analysis

Total proteins were extracted from cells using lysis buffer (Beyotime Biotechnology) supplemented with 1 mM of PMSF. Lysates were centrifuged at 12,000 rpm for 20 min at 4 °C. Thereafter, the supernatants were collected for protein concentration measurement using BCA™ Protein Assay Kit (PIERCE). Proteins (30 μg per sample) were separated by 12% or 15% SDS–PAGE and transferred to nitrocellulose membranes. Followed by blocking with 5% nonfat milk in TBST (0.1% Tween-20 in TBS) for 1 h at room temperature, the samples were probed with primary antibodies for overnight at 4 °C, and then with IRDye 680/800 labeled secondary antibodies for 1 h at room temperature after washing with TBST. The membrane was then examined by Odyssey Infrared Imaging System (LI-COR Biosciences).

The primary antibodies included rabbit anti-BRE (1:1000 dilution) and rabbit anti-phospho-Rb (Ser807/811) (1:1000) from Cell Signaling Technology; goat anti-PCNA (1:200), goat anti-CDK2 (1:200), goat anti-Cyclin A (1:200) and rabbit anti-Cyclin E (1:200) from Santa Cruz Biotechnology; and mouse anti-α-Tubulin (1:15000) from NeoMarkers. The secondary antibodies included IRDye 680LT donkey anti-rabbit IgG, IRDye 800CW donkey anti-mouse IgG and IRDye 800CW donkey anti-goat IgG (LI-COR Biosciences, 1:15000).

### Quantitative reverse transcription-PCR

Total RNA was isolated from cells using RNeasy Mini Kit (Qiagen) according to the manufacturer’s protocol. cDNAs were generated using RevertAid First Strand cDNA Synthesis Kit (Thermo Scientific) with oligo (dT)_18_ primers. Quantitative PCR was performed on ABI ViiA 7 Real Time PCR System (Applied Biosystems) using SYBR Green Master Mix (SYBR *Premix Ex Taq* II, TaKaRa). Primers were listed in Supplementary Table S1. The reaction was initiated by heating to 95 °C for 30 s, followed by 40 cycles of 95 °C for 5 s and 60 °C for 30 s, and ended with 95 °C for 15 s, 60 °C for 1 min, and 95 °C for 15 s. All the qPCR reactions were performed in triplicate. The expression levels of interested genes were normalized to GAPDH as internal control, and calculated by 2 ^−ΔΔCT^ method.

### Statistical analysis

The results were presented as mean ± SD where applicable. Graphs were plotted using GraphPad Prism 5.0 software. Statistical analysis was performed using IBM SPSS Statistics 19.0 software. Student’s t-test was used to determine the level of significance with *P-value*s < 0.05 considered as statistically significant.

## Additional Information

**How to cite this article**: Shi, W. *et al.* BRE plays an essential role in preventing replicative and DNA damage-induced premature senescence. *Sci. Rep.* **6**, 23506; doi: 10.1038/srep23506 (2016).

## Supplementary Material

Supplementary Information
