# Supplementary material for: BRE plays an essential role in preventing replicative and DNA damage-induced premature senescence
Source: Sci Rep. 2016 Mar 22;6:23506. doi: 10.1038/srep23506 (PMC4802329; doi:10.1038/srep23506)
Supplement: Supplementary Information [file srep23506-s1.pdf]

Supplementary Information

BRE plays an essential role in preventing replicative and DNA damage-induced premature senescence

Wenting Shi[1], Mei Kuen Tang[1], Yao Yao[1], Chengcheng Tang[1], Yiu Loon Chui[2], Kenneth Ka Ho Lee[1]*

1. Stem Cell and Regeneration Thematic Research Programme, School of Biomedical Sciences, Chinese University of Hong Kong, Hong Kong, People's Republic of China

2. Department of Chemical Pathology, Chinese University of Hong Kong, Hong Kong, People's Republic of China

* Corresponding author: Kenneth Ka Ho Lee

Methods

Transfection with siRNA in fibroblasts

WT fibroblasts were seeded into 4-well plates at a density of 4×10^4 cells/well and transfected with 20 pmol of BRE-siRNA or scrambled siRNA (Eurogentec) as control using Lipofectamine 2000 (Invitrogen) according to the manufacturer's instruction. The target sequence of BRE-siRNA was 'AACTGGACTGGTGAATTTTCA'. At 72 h after transfection, the cells were either left untreated or treated with 10 Gy of gamma irradiation, followed by SA-β-Gal staining 7 days afterwards as described in Methods. The knockdown efficiency was analyzed by either RT-qPCR or Western blotting.

Hydrogen Peroxide-induced premature senescence

WT and $BRE^{-/-}$ fibroblasts grown in 4-well plates were either left untreated or treated with 200 μM of H_2O_2 diluted in culture medium for 2 h. Cells were then washed with PBS and incubated in fresh medium. SA-β-Gal staining was performed 7 days afterwards as described in Methods.

Supplementary Figure. S1

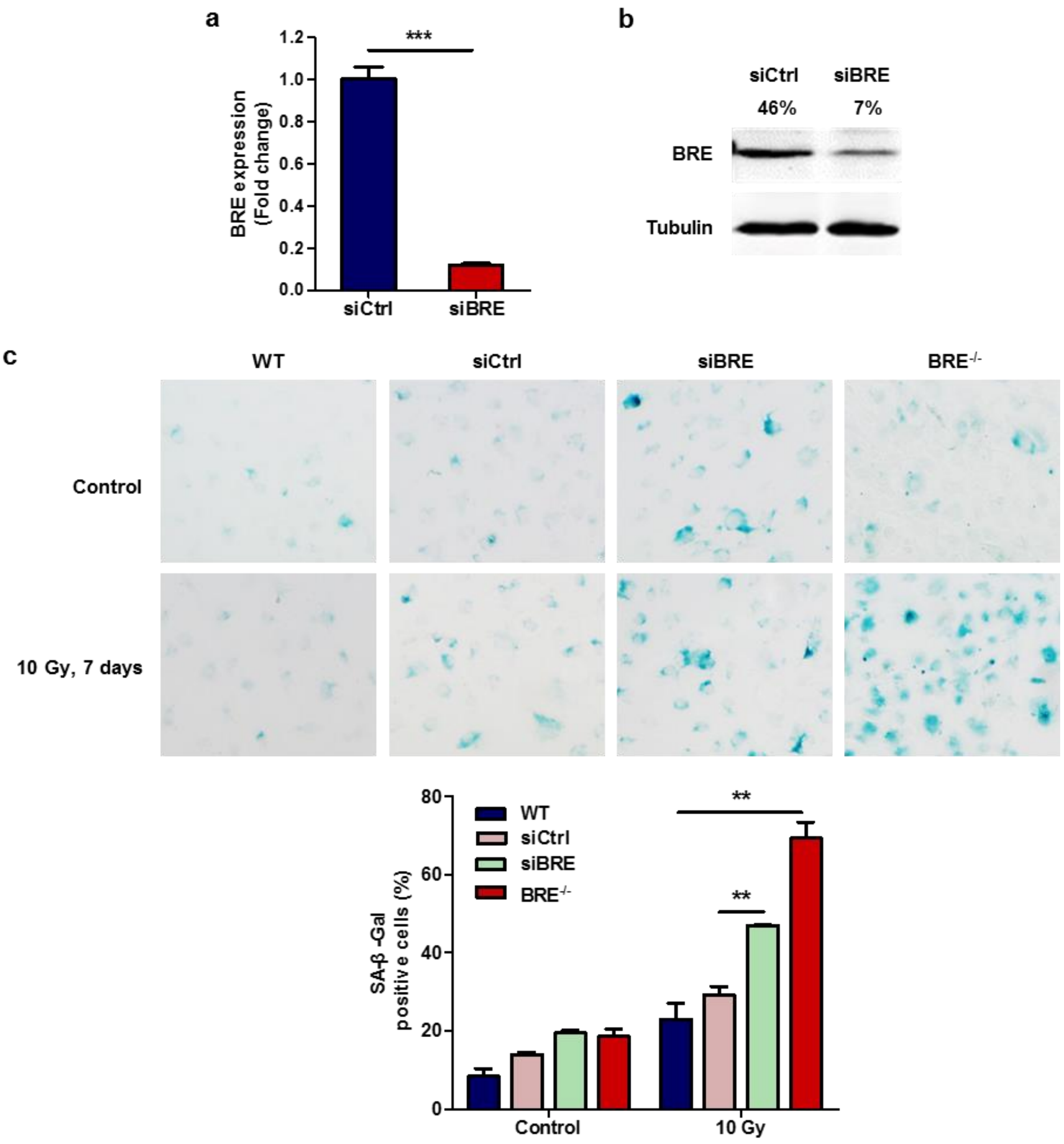

Supplementary Figure S1. BRE-silenced fibroblasts are more prone to DNA damage-induced premature senescence. (a) BRE mRNA expression in control and BRE-silenced fibroblasts as determined by RT-qPCR. House-keeping gene *GAPDH* was used for normalization. The data were analyzed using the comparative CT method and the fold change of gene expression was indicated as $2^{-\Delta\Delta CT}$. Data shown represent the mean ± SD of three independent experiments. *** denotes *P* value < 0.001 for the

difference between the control and BRE-silenced fibroblasts. **(b)** BRE protein expression in control and BRE-silenced fibroblasts by Western blotting with α-tubulin as protein loading control. The expression level of each protein relative to that of α-tubulin is shown as a percentage value on top of its respective image. **(c)** Senescence-associated β-Gal (SA-β-Gal) staining of WT, $BRE^{-/-}$, control and BRE-silenced fibroblasts at 7 days after 10 Gy of irradiation (top panel). The percentage of SA-β-Gal positive cells increased significantly between the irradiated $BRE^{-/-}$ fibroblasts compared with WT fibroblasts, also between the irradiated control and BRE-silenced fibroblasts (bottom panel). At least 500 cells were counted in no fewer than five fields of duplicate plates. Data represent as the mean ± SD. ** denotes P value < 0.01.

Supplementary Figure. S2

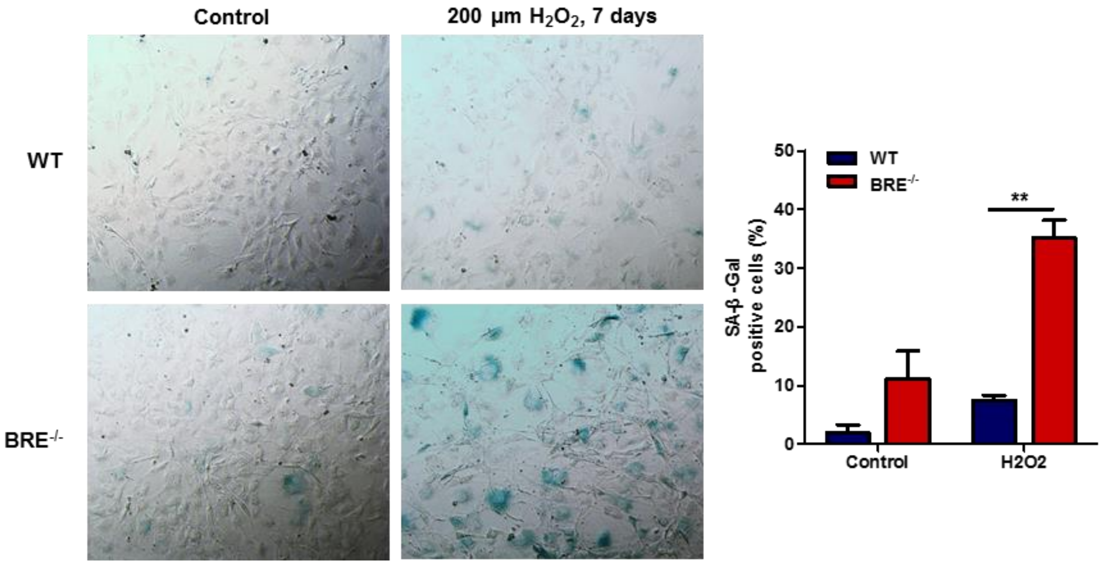

Supplementary Figure S2. BRE$^{-/-}$ fibroblasts are sensitized to H_2O_2 induced premature senescence. WT and BRE$^{-/-}$ fibroblasts were stained for SA-β-Gal activity on day 7 after after treatment with 200 μM of H_2O_2 (left panel). The SA-β-Gal positive cells among the WT and BRE$^{-/-}$ fibroblasts quantified in percentage values (right panel). At least 500 cells were counted in no fewer than five fields of duplicate plates. Data represent as the mean ± SD. ** $P < 0.01$ versus WT. Note the significant increase in SA-β-Gal positive cells among the BRE$^{-/-}$ versus WT fibroblasts.

Supplementary Figure. S3

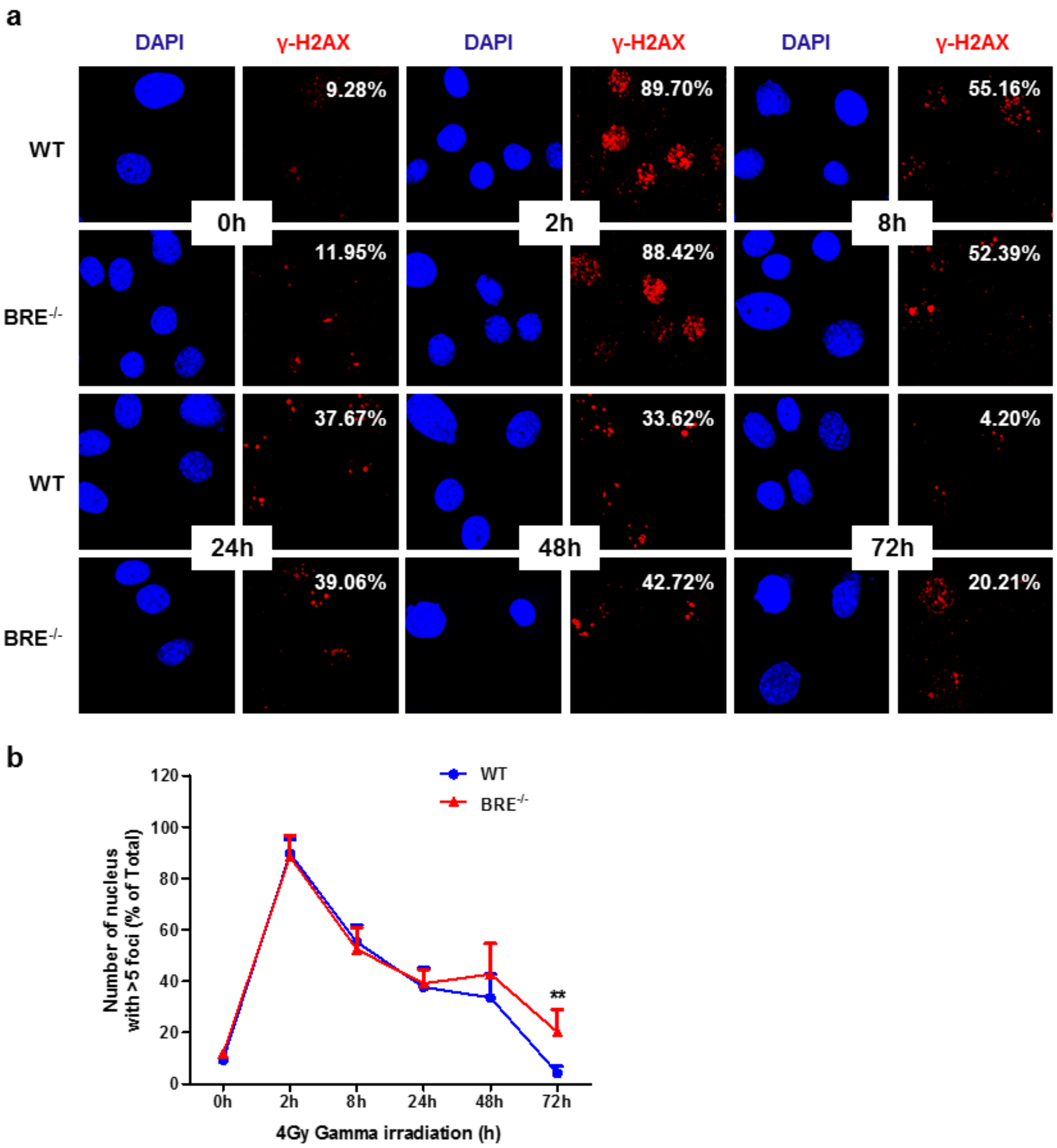

Supplementary Figure S3. The presence of γ-H2AX foci persists longer in BRE$^{-/-}$ fibroblasts compared with WT fibroblasts. (a) Immunofluorescence staining of passage 4 WT and BRE$^{-/-}$ fibroblasts at indicated time points after 4 Gy of gamma irradiation using anti-γ-H2AX antibody (red), together with DAPI nuclear staining (blue). **(b)** Percentage of nuclei with more than five γ-H2AX foci of the WT and BRE$^{-/-}$ fibroblasts above at the indicated time points. At least 150 cells were scored for the

γ-H2AX foci in no fewer than five fields of duplicate plates. Data represent the mean ± SD. ** $P < 0.01$ versus WT. Note the significantly longer persistence of γ-H2AX foci in $BRE^{-/-}$ fibroblasts compared with WT fibroblasts at 72 h.

Supplementary Table S1. Sequences of primers for RT-qPCR

Name	Direction	Primer sequence (5' to 3')
BRE	Forward	CCAGGTGTACCCCAAGTTGT
	Reverse	CAATGAGACATCCTCCTCCG
BRCA1	Forward	GCCGTCCAAATTCAAGAAGTA
	Reverse	TGGTCACACTTTGTGGAAACA
BARD1	Forward	TCTGTGGACCTCCAGGAACT
	Reverse	GGGATTCTCAGTGGATGCTG
BRCA2	Forward	AGGTTCAAAATTGTATGGGGG
	Reverse	TTGGGAAATTTTTAAGGCGA
RAD51	Forward	CCTCGCGCATATGCTACATT
	Reverse	ACACCGAGGGCACCTTTAG
BRCC36	Forward	CTGACAAGAGAAAGGACCGTG
	Reverse	CTCTCATGGGACGACCTGTT
MERIT40	Forward	CTGTATGACCTGGAGACGGC
	Reverse	GGGATGGTTTGCACATTCTC
RAP80	Forward	CCCACAAAGATTGAACAGCA
	Reverse	TGTTCTTGGCCTCTCTTCGT
ABRA1	Forward	CTGTATGACCTGGAGACGGC
	Reverse	GGGATGGTTTGCACATTCTC
GAPDH	Forward	CGTCCCGTAGACAAAATGGT
	Reverse	TTGATGGCAACAATCTCCAC
